# Aqueous Solution-Grown Crystalline Phosphorus Doped Indium Oxide for Thin-Film Transistors Applications

**DOI:** 10.3390/ijms232112912

**Published:** 2022-10-26

**Authors:** Wangying Xu, Tao Peng, Shuangmu Zhuo, Qiubao Lin, Weicheng Huang, Yujia Li, Fang Xu, Chun Zhao, Deliang Zhu

**Affiliations:** 1 Department of Physics, School of Science, Jimei University, Xiamen 361021, China; 2 College of Materials Science and Engineering, Shenzhen University, Shenzhen 518000, China; 3 Shenzhen Key Laboratory of Ultraintense Laser and Advanced Material Technology, Center for Advanced Material Diagnostic Technology, and College of Engineering Physics, Shenzhen Technology University, Shenzhen 518118, China; 4 Department of Electrical and Electronic Engineering, Xi’an Jiaotong-Liverpool University, Suzhou 215123, China

**Keywords:** thin-film transistors, solution-grown, crystalline oxide semiconductors, phosphorus doped indium oxide

## Abstract

Solution-grown indium oxide (In_2_O_3_) based thin-film transistors (TFTs) hold good prospects for emerging advanced electronics due to their excellent mobility, prominent transparency, and possibility of low-cost and scalable manufacturing; however, pristine In_2_O_3_ TFTs suffer from poor switching characteristics due to intrinsic oxygen-vacancy-related defects and require external doping. According to Shanmugam’s theory, among potential dopants, phosphorus (P) has a large dopant–oxygen bonding strength (E_M-O_) and high Lewis acid strength (L) that would suppress oxygen-vacancy related defects and mitigate dopant-induced carrier scattering; however, P-doped In_2_O_3_ (IPO) TFTs have not yet been demonstrated. Here, we report aqueous solution-grown crystalline IPO TFTs for the first time. It is suggested that the incorporation of P could effectively inhibit oxygen-vacancy-related defects while maintaining high mobility. This work experimentally demonstrates that dopant with high E_M-O_ and L is promising for emerging oxide TFTs.

## 1. Introduction

Transparent oxide semiconductors have emerged as promising materials for a wide range of applications, including thin-film transistors (TFTs) [1,2,3]; besides, oxide semiconductors could be solution-processed in an ambient atmosphere, showing great potential for scalable manufacturing [4,5,6,7,8]. In_2_O_3_ is a representative transparent oxide semiconductor with high mobility due to the large overlap of In 5 s orbital; however, the low binding energy of In-O (320.1 KJ/mol) favors oxygen-vacancy formation, resulting in high carrier density [9]. This often leads to high off-state-current and poor subthreshold swing when used as channel material for TFT applications. Previous investigations suggest that doping/alloying is an effective approach to solve this issue [10,11,12,13,14,15,16,17].

Based on Shanmugam’s theory, a promising dopant should have a large dopant–oxygen bonding strength (E_M-O_) and high Lewis acid strength (L) [11,12]. The large E_M-O_ is able to suppress oxygen-vacancy related defects; however, the introduction of extrinsic dopant would act as scattering centers, causing a drop in mobility [12]. The L can be described as: L = Z/r^2^ −7.7χ_z_ + 8.0, where Z represents the atomic nucleus charge number, r is the ionic radius, and χ_z_ designates the element electronegativity [12]. The high L could polarize electron charge away from the oxygen 2p valence band, weakening its activity as scattering centers and, hence, maintaining high mobility [11,12]; therefore, large E_M-O_ and high L are essential for the dopant element. Table 1 tabulated the E_M-O_ and L of common dopants for In_2_O_3_ host metal oxides [12]. Of all the potential dopants, phosphorus (P) has a large E_M-O_ (599.1 KJ/mol) and high L (10.082), showing great potential for controlling oxygen-vacancy related defects and alleviating dopant impurity carrier scattering. Unfortunately, P-doped In_2_O_3_ (IPO) TFTs have not yet been demonstrated and, hence, are worth investigating.

In this contribution, we first report the use of P as an efficient dopant for In_2_O_3_ TFTs. The IPO channel is fabricated via an aqueous solution route, which enables potential low-cost and expedient manufacturing. The impact of P incorporation is thoroughly investigated. The results show that the incorporation of P could effectively inhibit oxygen-vacancy-related defects while maintaining good mobility. The optimized IPO device (6% P) shows a decent mobility of 6.33 cm^2^/V s, threshold voltage of 2.97 V, subthreshold swing of 0.60 V/dec, and an excellent *I*_ON/OFF_ of ~10^8^, respectively.

## 2. Results and Discussion

**Figure 1:**
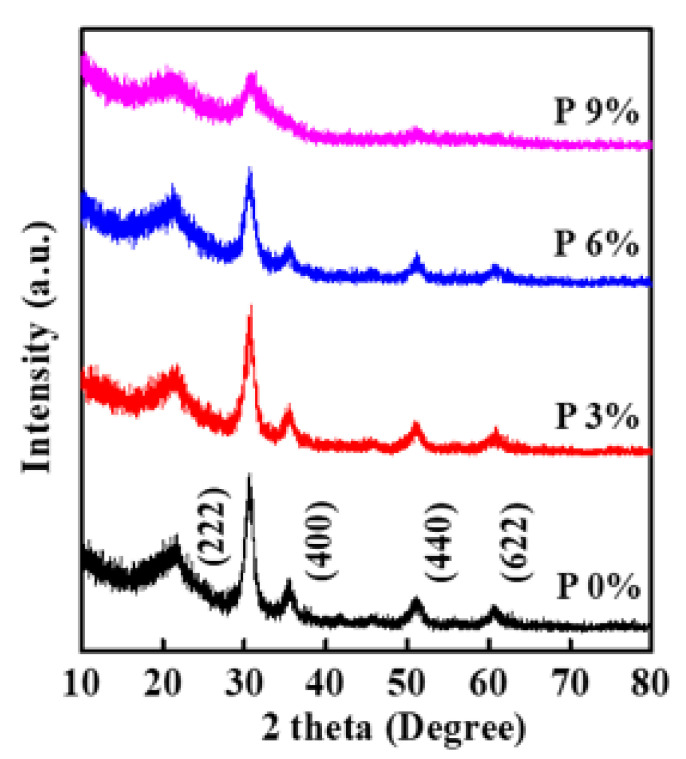
Grazing incidence XRD patterns for IPO films with indicated P ratio.

We performed grazing incidence XRD diffraction analysis on the crystalline structure of IPO films with different P proportions. As shown in Figure 1, the broad peaks at ~22.4° are attributed to the substrate. The characteristic diffraction peaks of (222) (dominant peak), (400), (440), and (622) are assignable to bixbyite In_2_O_3_ (JCPDS 06-0416). No P_2_O_5_ or other phases are observed from the diffraction pattern, which means that P addition does not break the In_2_O_3_ matrix crystalline structure [18,19]. We suppose that the added P replaces the In sites and maintains the cubic In_2_O_3_ structure when the P doping amount is relatively low; however, the diffraction peak intensity gradually decreases, suggesting the reduction of crystallinity after P incorporation [20]. This is because the P element would enhance the local distortions of the InO polyhedral and suppress the number of coordinate In atoms [13,20]. Previous studies also suggest that the addition of different-sized metal cations into the In_2_O_3_ could destroy the In_2_O_3_ lattice and reduce the crystallinity [10,11,13,20].

**Figure 2:**
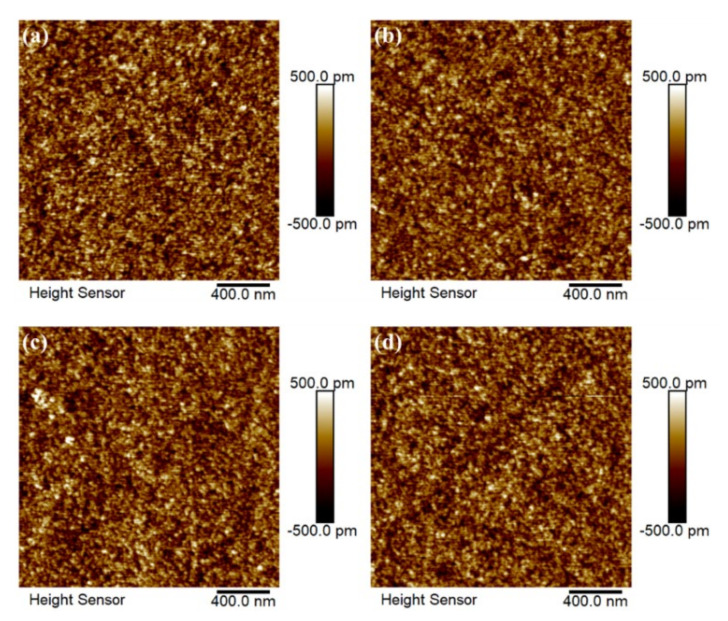
AFM images of the surface topography of IPO films with indicated P ratio of (**a**) 0%, (**b**) 3%, (**c**) 6%, and (**d**) 9%.

AFM measurements shown in Figure 2 indicate that the series of IPO films are highly smooth with RMS roughness of 0.27, 0.24, 0.31, and 0.26 nm for P ratio of 0, 3, 6, and 9%, respectively. The results suggest the absence of prominent grain boundaries in the crystalline IPO films and that P doping has little impact on the surface topography. It should be mentioned that the atomic smooth surface of the channel material is essential for transistor applications.

**Figure 3:**
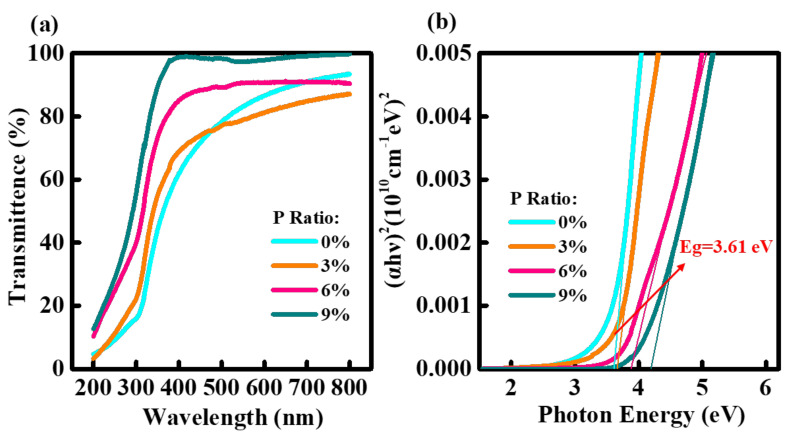
(**a**) Optical transmittance spectra of IPO films with various P ratios, and (**b**) corresponding optical bandgaps derived from the Tauc plots.

Figure 3a represents the optical transmittance spectra of IPO films with a range of P-doping contents. All the IPO films demonstrate excellent transparency (>79% at a wavelength of 550 nm). By using Tauc equation, the optical bandgaps of IPO films (Figure 3b) are calculated to be 3.61 eV (0% P), 3.66 eV (3% P), 3.88 eV (6% P), and 4.19 eV (9% P), respectively. The increased bandgap with P addition could be due to the fact that P can inhibit the formation of oxygen-vacancy defects in In_2_O_3_ [19]. The increased bandgap is more in line with the larger binding strength of P-O (599.1 KJ/mol) than that of In-O bonding (320.1 KJ/mol), indicating the effective oxygen-vacancy-related defects suppression [21].

**Figure 4:**
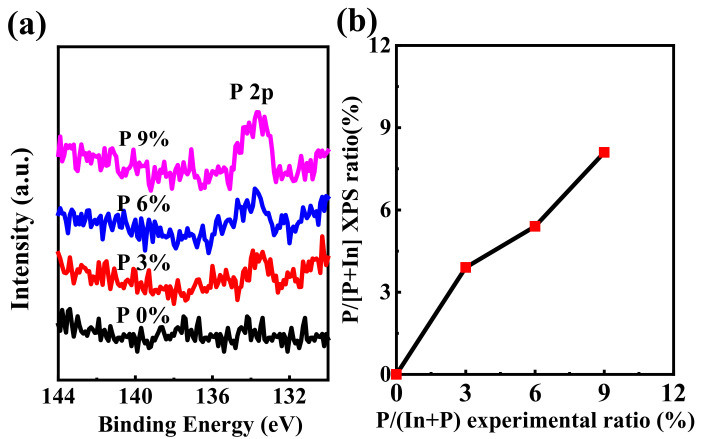
XPS spectra for the IPO films with different P-doping contents. (**a**) P 2p. spectra, and (**b**) P-doping ratio between solution and film.

XPS is supplemented for further verification. The corresponding P 2p spectra were demonstrated in Figure 4a. Previous reports have summarized the P 2p peak energies [22]. The peaks at ~135.6 eV, 133.5 eV, 128.6 eV, and 129.9 eV correspond to P_2_O_5_, phosphate, phosphide, and elemental P, respectively [22]; therefore, in the present work, the characteristic peak at ~133.5 eV is assigned to phosphate (PO_4_^3-^). In addition, as shown in Figure 4b, the P/In+ P ratio in the thin film steadily increases from 0 to 3.1, 5.6, and 8.4%, as the P ratio in the precursor increases from 0 to 3%, 6%, and 9%, respectively.

**Figure 5:**
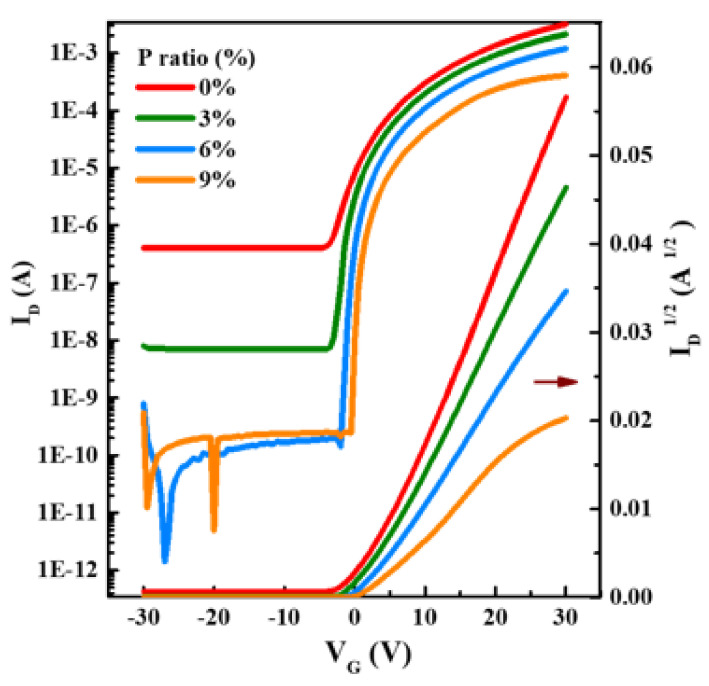
Transfer characteristics measured for IPO TFTs with different P ratios.

**Figure 6:**
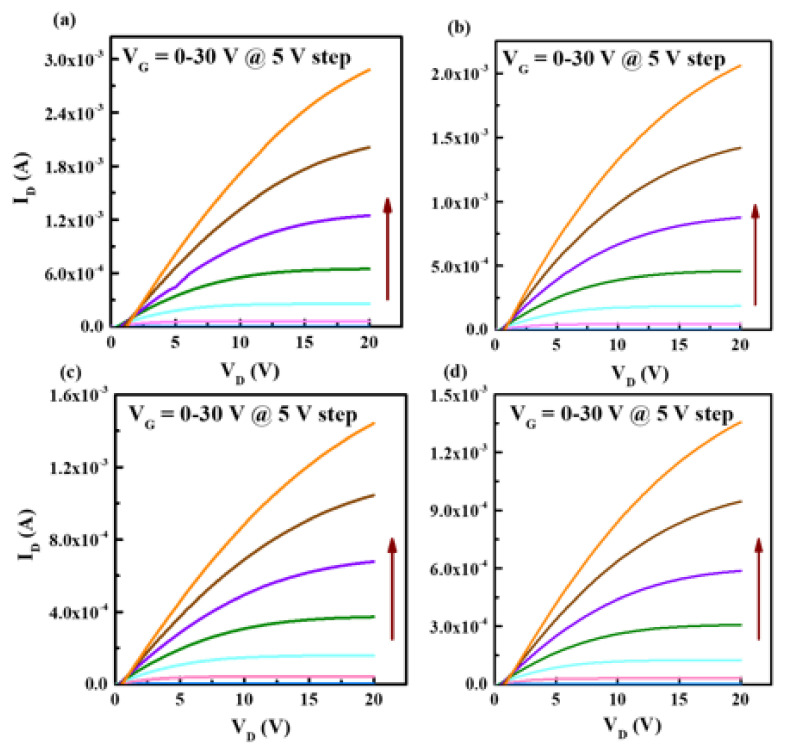
Output characteristics measured for IPO TFTs with P ratios of (**a**) 0%, (**b**) 3%, (**c**) 6%, and (**d**) 9%.

Figure 5 and Figure 6 show the representative transfer and output curves of the IPO TFTs with different P ratios, with electrical parameters summarized in Table 2. The pristine In_2_O_3_ TFT shows a good mobility of 16.2 cm^2^/V s, but high off-state current and poor subthreshold slope, which is typical behavior for an undoped-In_2_O_3_ device. The P incorporation leads to the great improvement of off-state-current and subthreshold swing, as well as positive shift of threshold voltage; besides, the trap density (including interface and bulk region, see Table 2) also improves after P incorporation, indicating that P doping weakens carrier scattering in IPO [23]. This is because the addition of P could inhibit the oxygen-vacancy-related defects due to the larger E_M-O_.

Unfortunately, P introduction would reduce the In 5 s orbital interaction near the conduction band, and, hence, decrease the mobility [10]; however, P has a high Lewis acid strength L, which could polarize electron charge away from the oxygen 2p valence band, weakening carrier scattering centers and, hence, relatively high mobility could be maintained [11,12]. The overall best IPO device (6% P) demonstrates a *μ* of 6.33 cm^2^/V s, *I*_on_/*I*_off_ of 1.1 × 10^8^, *S* of 0.60 V/dec, and V_th_ of 2.97 V, respectively, which is sufficient for transistor applications. The mobility of 6.33 cm^2^/Vs is relatively high since mobility larger than 5 cm^2^/V s is sufficient for liquid crystal display (LCD) applications (the mobility of commercial a-Si is 1 cm^2^/V s). Although P doping reduces the mobility of pristine In_2_O_3_, the other device parameters, especially on/off ratio, is greatly improved.

As shown in Table 3, we have provided the comparison with reported IGZO-related devices in the literature. Our newly designed IPO TFTs are among some of the best results. The newly designed IPO TFTs can be used in flat-panel displays and non-display applications, such as biosensors, photodetectors, memory, and neuromorphic devices.

## 3. Experimental Section

**Figure 7:**
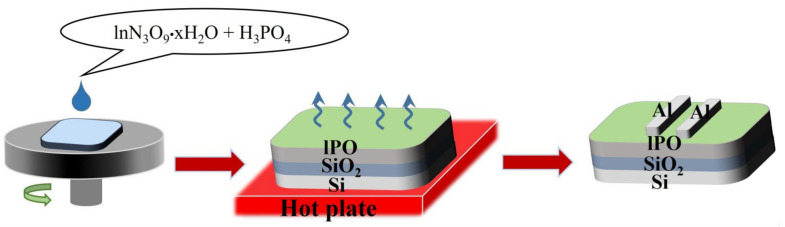
The IPO TFT device fabrication process.

Indium nitrate and phosphoric acid were dissolved in DI water with total concentration of 0.2 M and stirred for 2 h before deposition. The desired P/(In + P) atomic contents of 0, 3, 6, and 9% were produced. The IPO layers (~6 nm) were one-step spun at 4500 r.p.m. for 30 s on Si/SiO_2_ substrates and, then, thermally annealed at 300 °C for 120 min in ambient air. IPO TFTs (W/L = 1500/100 μm) fabrications were completed by evaporating Al source and drain electrodes. The IPO TFT device fabrication process is shown in Figure 7.

Grazing incidence XRD was performed on a Rigaku workstation (Austin, TX, USA). The surface topography information of IPO film was measured via AFM using Bruker Dimension Icon (Beijing, China). The chemical bonding states of IPO were analyzed via X-ray photoelectron spectroscopy (XPS, Thermo Microlab 350, Antigo, WI, USA). The transmittance information of the IPO layer was observed by UV-Vis spectrophotometry with PerkinElmer Lambda 950 (Waltham, MA, USA). Electrical current-voltage measurements of the transistors were carried out in air with a Keithley 2614B digital source meter (San Diego, CA, USA).

## 4. Conclusions

An efficient and tunable aqueous approach is reported to enhance In_2_O_3_ TFTs performance via P doping. It is suggested that P doping in In_2_O_3_ TFTs could suppress oxygen-vacancy-related defects that cause electron generation and trapping. The optimal IPO TFTs show enhanced switching characteristics. This work experimentally demonstrates that dopant with high dopant–oxygen bonding strength and Lewis acid strength is a promising candidate and opens a new way to fabricate high performance TFTs at low processing temperature.

## Figures and Tables

**Table 1:** Metal-oxygen bonding strength (E_M-O_) and Lewis acid strength (L) of elements.

Elements	Metal-Oxygen Bonding Strength (E_M-O,_ KJ/mol)	Lewis Acid Strength (L)
In^3+^	320.1	1.026
Ga^3+^	353.5	1.167
Ba^2+^	502.9	1.163
Mg^2+^	363.2	1.402
Al^3+^	511.0	3.042
Sb^5+^	434.3	3.559
P^5+^	599.1	10.082

**Table 2:** Summary of electrical properties of IPO TFTs investigated.

P Ratio (%)	Mobility (cm^2^/V s)	On/Off Ratio	Threshold Voltage (V)	Subthreshold Swing (V/dec)	Trap Densities (cm^−2^ eV^−1^)
0	16.2	3.8 × 10^3^	−0.57	2.52	8.6 × 10^12^
3	10.8	3.1 × 10^5^	1.98	0.97	2.9 × 10^12^
6	6.33	1.1 × 10^8^	2.97	0.60	1.4 × 10^12^
9	3.52	6.4 × 10^7^	3.27	0.55	1.2 × 10^12^

**Table 3:** Recent advances of solution-processed IGZO-related TFTs.

Channel Material	μ (cm^2^/Vs)	I_on_/I_off_	Ref
IZO	4	10^7^	[24]
IGZO	7.5	10^7^	[25]
IGZO	8.05	10^7^	[26]
IZO	2.43	10^7^	[27]
IGZO	1.38	10^5^	[28]
IGZO	2.64	10^8^	[29]
IPO	6.33	10^8^	This work
